# The Vaginal Microbiota: What Have We Learned after a Decade of Molecular Characterization?

**DOI:** 10.1371/journal.pone.0105998

**Published:** 2014-08-22

**Authors:** Janneke H. H. M. van de Wijgert, Hanneke Borgdorff, Rita Verhelst, Tania Crucitti, Suzanna Francis, Hans Verstraelen, Vicky Jespers

**Affiliations:** 1 Institute of Infection and Global Health, University of Liverpool, Liverpool, United Kingdom; 2 Academic Medical Center and Amsterdam Institute of Global Health and Development, Amsterdam, The Netherlands; 3 International Centre for Reproductive Health, Ghent University Hospital, Ghent, Belgium; 4 Prince Leopold Institute of Tropical Medicine, Antwerp, Belgium; 5 London School for Hygiene and Tropical Medicine, London, United Kingdom; 6 Department of Obstetrics and Gynecology, Ghent University Hospital, Ghent, Belgium; Fred Hutchinson Cancer Center, United States of America

## Abstract

We conducted a systematic review of the Medline database (U.S. National Library of Medicine, National Institutes of Health, Bethesda, MD, U.S.A) to determine if consistent molecular vaginal microbiota (VMB) composition patterns can be discerned after a decade of molecular testing, and to evaluate demographic, behavioral and clinical determinants of VMB compositions. Studies were eligible when published between 1 January 2008 and 15 November 2013, and if at least one molecular technique (sequencing, PCR, DNA fingerprinting, or DNA hybridization) was used to characterize the VMB. Sixty three eligible studies were identified. These studies have now conclusively shown that lactobacilli-dominated VMB are associated with a healthy vaginal micro-environment and that bacterial vaginosis (BV) is best described as a polybacterial dysbiosis. The extent of dysbiosis correlates well with Nugent score and vaginal pH but not with the other Amsel criteria. *Lactobacillus crispatus* is more beneficial than *L. iners*. Longitudinal studies have shown that a *L. crispatus*-dominated VMB is more likely to shift to a *L. iners*-dominated or mixed lactobacilli VMB than to full dysbiosis. Data on VMB determinants are scarce and inconsistent, but dysbiosis is consistently associated with HIV, human papillomavirus (HPV), and *Trichomonas vaginalis* infection. In contrast, vaginal colonization with *Candida* spp. is more common in women with a lactobacilli-dominated VMB than in women with dysbiosis. Cervicovaginal mucosal immune responses to molecular VMB compositions have not yet been properly characterized. Molecular techniques have now become more affordable, and we make a case for incorporating them into larger epidemiological studies to address knowledge gaps in etiology and pathogenesis of dysbiosis, associations of different dysbiotic states with clinical outcomes, and to evaluate interventions aimed at restoring and maintaining a lactobacilli-dominated VMB.

## Introduction

It has been known for some time that most vaginal microbiota (VMB) consist predominantly of lactobacilli and that VMB alterations can cause symptomatic conditions [1]. The most familiar condition is bacterial vaginosis (BV), which has traditionally been characterized as a reduction of vaginal lactobacilli and an overgrowth of other (facultative) anaerobic bacteria. In clinical settings, BV is typically diagnosed using Amsel criteria (three of the following four criteria should be present: 1) clue cells on wet mount microscopy; 2) a ‘fishy’ odour after adding 10% KOH to vaginal secretions; 3) vaginal pH>4.5; and 4) thin, homogenous vaginal discharge) [2]. In research settings, BV is also often defined by Gram stain Nugent scoring, which is based on microscopic visualization of three bacterial morphotypes (a Nugent score of 0–3 is considered normal, 4–6 intermediate microbiota, and 7–10 BV) [3]. BV is not highly inflammatory and is therefore often asymptomatic; this is why it is referred to as a vaginosis and not a vaginitis [1]. Two common types of microbiological vaginitis are vaginal candidiasis and trichomoniasis [1]. Vaginal candidiasis is often highly inflammatory and is typically diagnosed by wet mount microscopy and/or culture of *Candida* species. Trichomoniasis is caused by the sexually transmitted single-celled parasite *Trichomonas vaginalis*, which can be detected by microscopy, culture or PCR.

Other types of vaginitis have been described (such as aerobic vaginitis, desquamative inflammatory vaginitis, and atrophic vaginitis) but occur less frequently [4]–[6]. The term ‘aerobic vaginitis’ is used by some clinicians to refer to vaginal inflammation believed to be caused by streptococci, staphylococci and *Escherichia coli* [4]. While the roles of vaginal *Streptococcus agalactiae* (also known as Group B streptococcus) and *E. coli* in invasive maternal and neonatal infections have been well-documented [7], their potential roles in causing a vaginitis syndrome distinct from BV is not universally accepted.

Altered communities of micro-organisms in the vagina are not only implicated in septic postpartum and neonatal infections but also in pelvic inflammatory disease [8], miscarriage and pre-term birth [9], and increased HIV acquisition and onward transmission [10]–[12]. VMB alterations may therefore be of much greater public health importance than was previously assumed.

In the last decade, phylogenetic analyses of vaginal samples (mostly bacterial 16S ribosomal RNA gene sequencing) have shown that bacterial communities in the vagina are more complex than previously thought. The first study using molecular methods to characterize the VMB was published in 2002 [13]. In a review of studies conducted between 2002 and 2008, Srinivasan and colleagues concluded that BV is indeed associated with a loss of lactobacilli and the introduction and/or overgrowth of other (facultative) anaerobic bacteria, and identified important VMB bacteria that had previously been missed by culture-based methods [14]. These bacteria included species in the *Lactobacillus* genus (e.g. *L. iners*) and bacteria associated with BV (e.g. *Atopobium vaginae* and three bacteria in the *Lachnospiraceae* family temporarily named BVAB1, 2 and 3) [13], [15], [16].

Since 2008, high throughput molecular techniques have become more affordable and accessible, and many more VMB characterization studies have been performed. We conducted a systematic review of the published literature from 2008 to date, to synthesize current knowledge about the VMB and its determinants, and to identify research gaps.

## Methods

We conducted a systematic review according to the PRISMA 2009 guidelines [17].

Our first objective was to determine if any consistent VMB composition patterns can be discerned after a decade of molecular testing, despite the fact that different groups have used different molecular techniques and/or operating procedures. Our second objective was to review correlations between molecular compositions, Amsel criteria, and Nugent scoring. Our third objective was to assess which determinants (sociodemographic, physiological, and behavioral risk factors, and the presence of pathogens in the genital tract) have been consistently associated with certain VMB composition patterns in different studies.

### Search strategy and selection criteria

Eligible studies included studies that used at least one molecular technique (sequencing, PCR, DNA fingerprinting, or DNA hybridization). We only included PCR and DNA hybridization studies if multiple bacterial species or genera were assessed (either by multiple individual assays or by multiplex assays). We excluded studies that focused on viral, archaeal, fungal, or protozoal diversity, or on development of diagnostic assays. We only considered randomized controlled intervention trials if the baseline data, prior to the intervention, could be used to address one of our objectives. Article selection was based on the first objective; not all articles also addressed the second and third objectives.

We searched the Medline database (U.S. National Library of Medicine, National Institutes of Health, Bethesda, MD, U.S.A.) for articles between 1 January 2008 and 15 November 2013, limiting our search to articles written in English. We started our review in 2008 as opposed to 2002 (when the first molecular VMB data were published) because Srinivasan and Fredricks published a review of the early studies in 2008 [14]. We searched titles and abstracts using the search term ‘vaginal micr*’. Two authors (JvdW and HB) assessed the articles for eligibility, and hand-searched the reference lists of eligible articles to identify additional articles. Five authors (JvdW, HB, RV, VJ, and TC) extracted data from all eligible articles using predefined data extraction tables, which included the data categories presented in tables 1 and 2, a description of the VMB compositions and correlates that the study had identified, and study strengths and weaknesses. Each article was reviewed independently by two authors.

**Table 1:** Characteristics of molecular vaginal microbiota articles published between 1 January 2008 and 15 November 2013.

		Number ofarticles	References
**Year of**	2008	6	18–23
**publication**	2009	11	24–34
	2010	7	35–41
	2011	13	42–54
	2012	14	55–68
	2013^1^	12	69–80
	**TOTAL**	**63**	
**Country** 2	USA & Canada	31	19–21, 23–25, 27, 28, 30–32, 35–39, 42, 44, 49, 59–61, 64–70, 77, 78
	Belgium	6	26, 33, 34, 54, 62, 63
	Europe – other^3^	7	18, 22, 43, 55, 72, 76, 79
	China	6	29, 41, 47, 57, 74, 80
	Asia – other^4^	3	35, 50, 71
	South Africa	4	45, 46, 53, 73
	East Africa^5^	4	25, 40, 48, 77
	West Africa^6^	2	51, 58
	Central America^7^	3	52, 56, 75
	**TOTAL**	**66**	
**Sample size**	≤10	4	28, 29, 56, 70
**(women)**	11–50	22	18–20, 22, 23, 25–27, 31, 36–38, 43, 44, 48–50, 54, 59, 61, 69, 73
	51–100	18	24, 30, 33, 41, 45–47, 52, 55, 57, 58, 62, 63, 65, 66, 74, 78, 80
	101–200	14	21, 32, 34, 35, 39, 40, 53, 67, 68, 71, 75–77, 79
	>200^8^	5	42, 51, 60, 64, 72
	**TOTAL**	**63**	
**Study** **population**	Adults, healthy or with BV	28	19, 22, 23, 26, 28, 29, 35, 36, 38, 41–43, 50, 54, 57, 59, 60, 62–64, 67, 68, 70, 72, 73, 75, 79, 80
	Adolescents/virgins	3	24, 25, 66
	Pregnant women	7	33, 34, 52, 55, 58, 68, 78
	Postmenopausal women	5	43, 49, 55, 57, 71
	STD clinic attendees	7	19, 37, 38, 60, 62, 65, 76
	Female sex workers	2	25, 48
	WSW	3	21, 39, 61
	By HIV status	11	20, 30, 40, 44–46, 48, 51, 53, 58, 77
	By HPV/cytology status	5	46, 56, 66, 71, 74
	Women undergoing IVF	2	18, 69
	**TOTAL**	**78**	TOTAL
**Molecular** **technique** 2	NGS sequencing 454^9^	18	20, 36, 41, 42, 44, 48, 56, 58–60, 64, 65, 67, 68, 70–72, 76
	NGS sequencing other^9^	4	40, 49, 56, 67
	Culture & sequencing	15	18, 19, 23, 28, 29, 35, 50, 53, 54, 56, 57, 69, 73, 75, 78
	qPCR	19	21, 26, 30, 37–39, 41, 42, 47, 51, 55, 57, 61, 62, 63, 66, 77, 79, 80
	Multiplex PCR	4	27, 43, 51, 55
	Fingerprinting^10^	13	20, 24, 31, 33–36, 41, 43, 47, 52, 55, 74
	Hybridisation^11^	5	22, 25, 32, 45, 46
	**TOTAL**	**78**	
**Main topic** **of research** 2	Clustering of VMB	17	24, 28, 31, 35, 36, 40, 42, 48, 50, 56, 58, 60, 62, 65, 71, 72, 79
	Improving BV diagnosis	5	30, 47, 76, 79, 80
	Descriptive – other^12^	27	18, 21, 24, 28, 33, 35, 38, 39, 42, 48, 51, 54–56, 59, 60, 62, 63, 66, 68–70, 72, 73, 77–79
	Longitudinal VMB changes	20	18, 21, 23, 25, 26, 33, 38, 39, 42, 49, 54, 56, 59, 62, 63, 66, 67, 69, 77, 78
	Extravaginal reservoirs^13^	6	34, 43, 47, 55, 56, 61
	VMB associations with HIV	11	20, 30, 40, 44, 45, 46, 48, 51, 53, 58, 77
	VMB associations with HPV	5	46, 56, 66, 71, 74
	VMB associations with other STIs	4	51, 64, 72, 79
	VMB associations with other infections^14^	5	27, 32, 47, 65
	VMB associations with non-infectious reproductive health outcomes	4	32, 49, 69, 78
	VMB associations with immune activation	1	22
	**TOTAL**	**105**	

BV = bacterial vaginosis; DGGE = Denaturing Gradient Gel Electrophoresis; HIV = human immunodeficiency virus; HPV = human papillomavirus; IVF = in-vitro fertilisation; LH-PCR = Length Heterogeneity PCR; NGS = next generation sequencing; qPCR = quantitative polymerase chain reaction; Randomly Amplified Polymorphic DNA (RAPD); STD clinic = sexually transmitted disease clinic; STI = sexually transmitted infection; TRLFP = Terminal Restriction Fragment Length Polymorphism; USA = United States of America; VMB = vaginal microbiota; WSW = women having sex with women.

1 Until 15 November 20131;

2 One article could include more than one category;

3 Europe other: Sweden (4), Greenland (1), Estonia (1), Austria (1);

4 Asia other: Japan (2), South Korea (1);

5 East Africa: Kenya (3), Tanzania (1);

6 West Africa: Burkina Faso (1), Ghana, Togo, Guinea and Mali (1);

7 Central America: Mexico (2), Costa Rica (1);

8 5 publications representing only 3 studies;

9 Genes sequenced: 16S rRNA gene V1–V2 (8), V2–V3 (2), V3 (2), V3–V5 (3), V4–V6 (2), V6 (2), V6–V9 (1), chaperonin gene (1), and not reported (1);

10 Fingerprinting techniques used: TRFLP (5), DGGE (5), RAPD (2), and LH-PCR (1);

11 Hybridisation techniques used: DNA microarray (4) and Luminex (oligonucleotides coupled to beads; 1);

12 Descriptive other includes effects of race/ethnicity, pregnancy, menstrual cycle, menopause, tampon/pad use, vaginal douching, sexual debut and other sexual and contraceptive behaviours;

13 Cervical or endometrial microbiota (3) and oral and/or rectal reservoirs (3);

14 Other confirmed infections: Candidiasis (2), cervicitis (1), endometritis (1), and gingivitis (1).

**Table 2:** Vaginal microbiota communities identified by clustering techniques in 17 articles.

Cluster	Molecular techniques^1^	References	Total
Dominated by *Lactobacillus crispatus*	All except qPCR^2^	24, 31, 35, 36, 40, 42, 50,56, 65, 71, 72	11
Dominated by *L. iners*	All	24, 31, 35, 36, 40, 42, 48,50, 56, 58, 60, 65, 71, 72, 79	15
Dominated by *L. jensenii*	Direct sequencingand fingerprinting	35, 42	2
Dominated by *L. gasseri*	Direct sequencing and fingerprinting	31, 35, 36, 42, 56	5
Dominated by lactobacillibut unspecified	Sequencing of culture colonies	28	1
Dominated by lactobacillibut multiple species	All	24, 35, 60, 62, 65	5
Dominated by *Gardnerella vaginalis*	Direct sequencing and qPCR	40, 48, 56, 79	4
Mixture of lactobacilliand *G. vaginalis*	Direct sequencing andsequencing of culture colonies	28, 40, 56	3
Mixture of lactobacilli, *G. vaginalis*and other anaerobes^3^	All except qPCR	28, 31, 42, 48, 56, 58, 60, 65	8
Mixed anaerobes^3^ withfew/no lactobacilli	All	24, 28, 35, 36, 40, 50,56, 58, 60, 62, 71, 72, 79	13
Dominated by aerobes^4^	Direct sequencing and fingerprinting	35, 58, 65	3

qPCR = quantitative polymerase chain reaction.

1 Includes direct sequencing (next generation sequencing) of DNA extracted from vaginal samples (10), sequencing of culture colonies (2), fingerprinting (3), and qPCR (2); the 5 studies using DNA hybridisation techniques did not employ data clustering and this technique is therefore not represented in this table.

2 One qPCR study only assessed *L. iners* and the other qPCR study did not find clusters dominated by just one *Lactobacillus* species.

3 Other than *Lactobacillus* spp., the 25 most abundant taxa in the 10 direct sequencing studies consistently (in at least 50% of studies) include: Phylum Actinobacteria: *G. vaginalis*, *A. vaginae*, *Eggerthella* spp., *Mobiluncus* spp.; Phylum Fermicutes: *Lachnospiraceae* (including BVAB1-3), *Dialister* spp., *Megasphaera* spp., *Parvimonas* (formerly *Peptostreptococcus*) spp., *Veillonella* spp., *Streptococcus* spp., *Staphylococcus,* spp., *Gemella* spp.; Phylum Sfingobacteria: *Prevotella* spp., *Porphyromonas* spp., *Bacteroides* spp.; Phylum Fusobacteria: *Sneathia* spp., *Leptotrichia* spp.; Phylum Tenericutes: *Mycoplasma* spp., *Ureaplasma* spp.; Phylum Proteobacteria: *Escherichia/Shigella* spp.

4 Includes *Streptococcus* spp., *Staphylococcus* spp., *Escherichia/Shigella* spp., *Proteus* spp.

## Results

### Study selection

Our Medline database search yielded 475 results, of which 50 were eligible. We identified 20 additional eligible articles from the reference lists of the initial 50 articles. After data extraction, a further seven articles were rejected because they did not address our objectives appropriately (mostly because they focused on technical laboratory issues or diagnostic assay development). A total of 63 articles are therefore included in this review [18]–[80].

### Study characteristics

Table 1 shows characteristics of the 63 articles (references are included in the table and are not repeated here). It should be noted that one article could include data from more than one data extraction category, which is why some column totals exceed 63. Most of the articles reported data from North America (31 articles), followed by Europe (13), Africa (10), Asia (9), and Central America (3). Most sample sizes were small, with only 19 articles reporting a sample size larger than 100. Most of the study populations were non-pregnant adult women of reproductive age, with or without BV as the only diagnosed condition (28 articles). Other study populations included adolescents/virgins (3 articles), pregnant women (7), postmenopausal women (5), women attending a sexual health clinic (7) or with confirmed HIV, HPV or other infections (16), female sex workers (2), women who have sex with women (WSW; 3), and women undergoing in-vitro fertilization (IVF) (2). Sequencing was the most commonly employed molecular technique; earlier studies tended to sequence DNA isolated from individual colonies obtained by bacterial culture (15 articles), whereas later studies extracted DNA directly from genital samples followed by next generation sequencing (18 studies used 454 sequencing (454 Life Sciences Corporation, Branford, CT, US) and four used other platforms). PCR was also commonly used with 19 studies using quantitative PCR (qPCR) for individual species/genera, and four studies using qualitative multiplex PCR. Other molecular techniques included DNA fingerprinting techniques (13), phylogenetic DNA microarrays (4), and hybridization to oligonucleotide probes coupled to beads (1). One of the main aims in all articles was to describe the VMB in a particular study population, and 20 articles included longitudinal data (indicated in Table 1 as longitudinal VMB changes); three articles also described cervical and/or endometrial microbiota and three rectal and/or oral reservoirs of vaginal bacteria; and 17 employed a clustering technique to characterize bacterial communities.

### Definitions used for a healthy VMB and for BV

Most of the 63 articles that we reviewed used a Nugent score of 0–3 to define a healthy VMB (26 articles), with an additional 12 using a Nugent score of 0–3 plus the presence of fewer than three Amsel criteria, and nine using a Nugent score of 0–6. Fourteen articles did not provide a definition, and the remaining 27 articles used a variety of definitions based on microscopy, vaginal pH, and clinical symptoms. In the 37 articles that described a comparison between a healthy VMB and BV, BV was mostly defined as a Nugent score of 7–10 (20 articles), with an additional nine using a Nugent score of 7–10 plus the presence of three or more Amsel criteria, and the remaining eight using a variety of other definitions based on microscopy and clinical symptoms.

### Vaginal bacterial communities by clustering

The 17 studies that used a clustering technique to characterize the composition of VMB bacterial communities can be subdivided into those that used comprehensive data based on next generation sequencing of DNA extracted from vaginal samples (10 articles) [36], [40], [42], [48], [56], [58], [60], [65], [71], [72] and those that used less comprehensive data by sequencing DNA of bacterial culture colonies (2) [28], [50], fingerprinting (3) [24], [31], [35], or qPCR (2) [62], [79] (Table 2). The articles included different study populations and employed a variety of molecular and clustering procedures, but consistent clustering patterns can be discerned nonetheless. A total of three to nine clusters were described. The majority of studies found one cluster dominated by *L. iners* (15 articles) and one dominated by *L. crispatus* (11) (Table 2). Clusters dominated by *L. jensenii* (2 articles), *L. gasseri* (5), or *G. vaginalis* (4) were less common. Clusters containing high proportions of multiple *Lactobacillus* spp., or *Lactobacillus* spp. combined with *G. vaginalis,* were described in eight articles.

All studies identified at least one cluster that was not dominated by a single taxon, but contained mixtures of anaerobes with or without *Lactobacillus* spp. (Table 2). These clusters typically contained *L. iners* (sometimes *L. gasseri*) and *G. vaginalis*, plus mixtures of other strict or facultative anaerobic bacteria. In the ten studies that employed next generation sequencing of DNA extracted from vaginal samples, the 25 most abundant taxa consistently (in at least 50% of studies) included the following additional taxa: *A. vaginae*, *Eggerthella* spp., *Mobiluncus* spp., *Lachnospiraceae* (including the species BVAB1-3), *Dialister* spp., *Megasphaera* spp., *Parvimonas* (formerly *Peptostreptococcus*) spp., *Veillonella* spp., *Streptococcus* spp., *Staphylococcus* spp., *Gemella* spp., *Prevotella* spp., *Porphyromonas* spp., *Bacteroides* spp., *Sneathia* spp., *Leptotrichia* spp., *Mycoplasma* spp., *Ureaplasma* spp., and *Escherichia/Shigella* spp. Other bacterial taxa were often found but either in low abundance or not consistently. Most articles identified more than one cluster with mixed taxa; these clusters typically did not differ significantly in the total number or types of bacterial taxa present but they did differ in their relative proportions. We were not able to discern consistent patterns across studies. Only three of the 17 articles reported clusters dominated by streptococci, staphylococci, *Proteus* spp., or *Escherichia*/*Shigella* spp. (Table 2).

### Longitudinal VMB patterns

One of the conclusions of the Human Microbiome Project was that within-subject microbiota variation over time was lower than between-subject variation for all habitats, including the vagina [67]. Similarly, several longitudinal VMB studies showed that a majority of women have a stable VMB microbiome [23], [26], [54], [59], [62]. A study in post-menopausal women showed that the VMB is usually stable in that group as well [49]. However, while one study suggests that an increased VMB diversity is associated with a decreased stability [23], others suggest that this is not necessarily the case: for example, a BV-associated VMB can be stable and persist for a long time [59]. The few articles that describe VMB transitions in molecular detail agree that women who have a *L. crispatus*-dominated VMB at baseline are less likely to transition to a BV-associated VMB than women who have a *L. iners*-dominated VMB [33], [30], [59]. The study that evaluated VMB transitions in the greatest detail found that a *L. crispatus* VMB more often transitions to a *L. iners*-dominated or mixed lactobacilli VMB than to a BV-associated VMB, and that a *L. iners*-dominated VMB compared to a *L. crispatus*-dominated VMB is twice as likely to transition to a BV-associated VMB [59]. Data on transitions related to *L. jensenii*, *L. gasseri*, and *L. vaginalis*-dominated VMBs are rare [33], [59]. One study in women who had clinical BV showed that BV is more likely to be persistent when BVAB1-3, *Peptoniphilus lacrimalis*, or *Megasphaera* type 2 are present in the VMB [21].

### Extravaginal reservoirs of VMB bacteria

The Human Microbiome Project also concluded that vaginal microbial communities are relatively ‘simple’ at genus-level compared to oral and gut communities, but have a higher diversity of *Lactobacillus* spp. [67]. Solt *et al*. identified 673 genera in the rectum, 275 in the mouth, and 112 in the vagina [81]. Three studies assessed the presence of lactobacilli and other VMB taxa in rectal and oral specimens, as well as vaginal specimens, to test the hypothesis that the gut and mouth act as extravaginal reservoirs of VMB bacteria [34], [55], [61]. Lactobacilli and various BV-associated bacteria were indeed often found in the rectum, while lactobacilli were sometimes, and *G. vaginalis* consistently, found in the mouth. In one study, women who had high quantities of *G. vaginalis* in the mouth or rectum, or *Megasphaera*, *Leptotrichia*, or *Sneathia* spp. in the rectum, were more likely to develop clinical BV during follow-up; in contrast, women who had *L. crispatus* in the rectum were less likely to develop clinical BV [61].

### VMB associations with traditional BV diagnostics

More than half of the articles (37) described molecular VMB data in relation to BV diagnosis by Amsel and/or Nugent criteria [19]–[23], [25]–[27], [30], [32], [33], [37], [38], [40]–[42], [45], [47], [48], [50]–[54], [56], [59], [60], [62], [63], [65], [67], [72], [73], [75], [76], [79], [80]. Two important areas of consensus emerged. First, almost all women carry vaginal lactobacilli regardless of their BV status by Nugent or Amsel criteria [19], [20], [22], [23], [25], [26], [33], [37]–[42], [45], [47], [48], [54], [59], [60], [62], [63], [73], [76], [80] but *L. crispatus* is predominantly found in BV-negative women [20], [22], [23], [25], [32], [33], [37], [38], [40], [42], [48], [50], [54], [59], [60], [62], [63], [65], [73], [75], [76], [80] whereas *L. iners* (and to a lesser extent *L. gasseri*) is also found in women with intermediate microbiota or BV [21], [23], [25], [33], [37], [38], [40]–[42], [47], [50], [54], [59], [60], [62], [63], [65], [73], [76], [79], [80]. This is also reflected in correlation studies, in which *L. iners* correlates well with BV-associated bacteria but not with *L. crispatus* [56], [65]. A second area of consensus is that BV diagnosis is characterized by increased bacterial diversity and the presence of the multiple taxa of (facultative) anaerobes that were described in the previous paragraph [19]–[23], [25]–[27], [30], [32], [33], [37], [38], [40]–[42], [45], [47], [48], [50]–[54], [56], [59], [60], [62], [63], [65], [67], [72], [73], [75], [76], [79], [80]. qPCR studies consistently report a decline in overall *Lactobacillus* load in BV, and an increase in bacterial loads of BV-associated bacteria [26], [41], [47], [62], [76], [80]. It is not yet clear from the studies we reviewed whether BV is associated with a higher overall bacterial load than healthy lactobacilli-dominated microbiota. Importantly, several studies showed that *G. vaginalis* and *Prevotella* spp. are often found regardless of BV status by Nugent or Amsel criteria, but their abundances increase in BV; furthermore, a synergistic effect between them was noted [26], [37], [41], [42], [62], [79]. The role of streptococci, staphylococci, and enterococci is generally not well described; when present, they are usually present in low abundance. One study (using DNA hybridization) reported that their presence in the VMB did not differ by BV status [32], whereas another study (also using DNA hybridization) reported higher levels in women with intermediate microbiota by Nugent score [22].

Higher bacterial diversity and/or higher levels of individual BV-associated bacteria are consistently associated with a higher vaginal pH [30], [42], [60], [67], [72], [80] and Nugent score [42], [47], [51], [80]. Vice versa, increasing abundance of lactobacilli is consistently associated with a lower vaginal pH [30], [40], [67], [80] and Nugent score [80]. Associations with the other Amsel criteria have been less well studied, but one study found that only the *Leptotrichia* and *Eggerthella* genera were associated with all four Amsel criteria [60].

### VMB associations with other clinical outcomes

Vaginal colonization with *Candida* spp. seems more common in women with a lactobacilli-dominated VMB than in women with BV [26], [31], [72], as had been noted previously in epidemiological studies using traditional diagnostic methods [11]. In contrast, *T. vaginalis* has often been strongly associated with BV in the past, and this was confirmed in a study using 454 sequencing [64]. Convincing patterns of associations between bacterial sexually transmitted infections (STIs) and the molecular composition of the VMB did not emerge, most likely due to the fact that women with bacterial STIs were often excluded or the prevalence rates were low.

Eleven studies assessed the VMB by HIV status [20], [30], [40], [44]–[46], [48], [51], [53], [58], [77] and five studies by HPV status [46], [56], [66], [71], [74]. One study found no relationship between HIV and the VMB [53], but most found trends towards decreased lactobacilli (and particularly *L. crispatus*) [44], [46], [48] and increased bacterial diversity [45], [51], particularly in women who had both HIV as well as BV by Nugent score [20]. Similar trends were found related to HIV-1 RNA load in the genital tract [30], [77]. One study found an increased prevalence of *E. coli*-dominated VMB in HIV-positive women [48], and another one found increased HIV transmission from mother to child with increasing VMB diversity in the mother (although this did not reach statistical significance) [58]. HPV also seems to be associated with a reduction in lactobacilli [71] and increased VMB diversity [46], [71], [74]. In one study, *Sneathia* spp. were strongly associated with the presence of high risk HPV [71].

The cervical microbiota are similar to the VMB, except that bacterial loads are lower [47]. A comprehensive qPCR study showed that cervical bacterial diversity is highest in women with BV, followed by women with cervicitis and healthy women, with only small differences between the latter two; BV was associated with a dramatic reduction in lactobacilli in the vagina and cervix, whereas cervicitis with a reduction in the cervix only [47]. The authors conclude that the VMB does not play a large role in cervicitis. Another study found good agreement between PCR results of five BV-associated species in cervical and endometrial samples of women with pelvic inflammatory disease, although this did not reach statistical significance [27].

BV and gingivitis were also reported to be associated, with counts of *P. bivia, P. disiens, M. curtisii,* and *M. mulieris* being particularly high in women with both BV and gingivitis [32]. Finally, a lactobacilli-dominated VMB was associated with a reduced risk of pre-term birth, a higher likelihood of IVF resulting in a live birth, and a reduced risk of vaginal dryness in postmenopausal women in one study each [49], [69], [78].

We found only one study that correlated molecular VMB composition (using both culture and a DNA-DNA checkerboard including *L. iners* and 12 BV-associated species) with vaginal immune responses [22]. Total viable bacterial counts and the presence of BV-associated bacteria were positively associated with cervicovaginal IL-1α and IL-1β (and BV-associated bacteria also with IL-6 and IL-8), whereas *L. iners* was negatively associated with IL-1α. The relationships with secretory leukocyte protease inhibitor (SLPI) were the other way around.

### VMB associations with demographic and behavioral characteristics

Data on the association between the molecular VMB and age were inconsistent. Six studies did not find an association but four of these only included a narrow age range (exclusively reproductive age or post-menopausal women) [42], [49], [62], [72]; one study did not find a difference between reproductive age and post-menopausal women [76] and another one did not find a difference between adolescents and women of reproductive age [24]. However, three studies that quantified multiple *Lactobacillus* spp. found lower overall levels, as well as reduced *L. crispatus* levels and *Lactobacillus* diversity, in post-menopausal women compared to women of reproductive age [43], [55], [57].

Several articles report that Black African and African-American women compared to Caucasian or Asian women are less likely to carry *L. crispatus, L. jensenii*, *L. gasseri* and/or *L. vaginalis* and more likely to carry *L. iners*, and are more likely to have a higher bacterial diversity [35], [42], [60], [62], [77], [78]. One study found the same for U.S. Hispanic women [42].

Few studies found significant associations between the VMB at the molecular microbial level and sexual behavior. However, detailed sexual behavior data were mostly not collected, sample sizes were small, or analyses focused on risk factors for BV by Amsel or Nugent criteria even though bacterial molecular data were also available. One study found that the detection of prostate-specific antigen (as a marker of sexual activity within the last 48 hours) was negatively associated with *L. crispatus* and positively with *L. iners* and *L. gasseri* [62]. In another study, the prevalence of various BV-associated bacterial genera was increased with an increasing number of sexual partners [51]. Finally, a comprehensive study found a slight gain of *G. vaginalis* after sexual debut, but no significant gain of other BV-associated bacteria or loss of lactobacilli [66].

Even though most studies that evaluated the influence of the menstrual cycle were small, they consistently suggest that high levels of estradiol (assessed by phase in the menstrual cycle or in serum of IVF patients) promote lactobacilli, and particularly *L. crispatus* [18], [59], [69], [70]. Studies also consistently suggest that menses is the largest disturbing factor during the menstrual cycle, with sometimes large reductions in lactobacilli [38], [59], [62], [63], shifts from *L. crispatus* to *L. iners* [38], [70], or the appearance of BV-associated bacteria, streptococci or other Gram-positive cocci [54], [70]. Pregnancy, which is also accompanied by high estradiol levels, is associated with high levels of lactobacilli, particularly *L. crispatus*, and low bacterial diversity [55], [68]. However, one study found an increasing bacterial diversity in late term pregnancies [68]. In another study, a VMB dominated by *L. iners* or *L. gasseri* in the first trimester was more likely to evolve to BV later on during pregnancy; *L. crispatus* had the opposite effect [33].

## Discussion

Despite the fact that many different molecular techniques and operating procedures with specific advantages and disadvantages have been used (reviewed in [14]) and despite the fact that these technical differences can result in under- or overrepresentation of bacterial species [82], we found several areas of consensus about the VMB composition. Studies have now conclusively shown that lactobacilli-dominated VMB are associated with a healthy vaginal micro-environment, and that BV is best described as a polybacterial dysbiosis: the *Lactobacillus* load decreases, and both the diversity and bacterial load of other (facultative) anaerobic bacteria increase [24], [28], [31], [35], [36], [40], [42], [48], [50], [56], [58], [60], [62], [65], [71], [72], [79]. Furthermore, the bacteria associated with this dysbiosis are now well described [24], [28], [31], [35], [36], [40], [42], [48], [50], [56], [58], [60], [62], [65], [71], [72], [79]. Some are consistently found (*G. vaginalis*, *A. vaginae*, bacteria in the *Lachnospiraceae* family (including BVAB1-3), and species in the following genera: *Prevotella*, *Eggerthella*, *Dialister*, *Megasphaera*, *Sneathia*, *Leptotrichia*, *Parvimonas* (formerly *Peptostreptococcus*), *Veillonella*, *Bacteroides*, *Mobiluncus*, *Porphyromonas*, *Mycoplasma*, *Ureaplasma*, *Streptococcus*, *Staphylococcus, Gemella*, and *Escherichia/Shigella*) whereas others are not consistently found but can be part of a long tail of minority species. *G. vaginalis* and *Prevotella* spp. are also often present in healthy women, but their bacterial loads increase significantly in dysbiosis. Consensus is also emerging about the relative importance of different *Lactobacillus* species: *L. iners* is present in almost all women worldwide including those with dysbiosis; *L. crispatus* is mostly present in healthy women and might be less common in women of African or Hispanic descent; and *L. jensenii*, *L. gasseri*, and *L. vaginalis* are much less common [24], [28], [31], [35], [36], [40], [42], [48], [50], [56], [58], [60], [62], [65], [71], [72], [79]. Furthermore, longitudinal studies have shown that a *L. crispatus*-dominated VMB might transition to a *L. iners*-dominated VMB but is less likely to transition directly to a dysbiotic state (and vice versa) [33], [59]. The gut, and to a lesser extent the mouth, serve as extravaginal reservoirs of common VMB bacteria [34], [55], [61].

An increase of bacterial diversity and BV-associated bacteria is consistently associated with an increase in Nugent score and/or vaginal pH, but not with the other three Amsel criteria [30], [42], [47], [51], [60], [67], [72], [80]. This is reassuring because our current knowledge about the epidemiology of vaginal dysbiosis is mostly based on Nugent scoring. A recent study by Srinivasan and colleagues, however, questioned the microbial interpretation of Nugent scoring [83]. This study showed that the *Mobiluncus* morphotype more likely represents BVAB-1 than *Mobiluncus* spp., and the *Bacteroides* morphotype more likely represents *Porphyromonas* and *Prevotella* spp than *Bacteroides* spp. While these are important observations, the clinical relevance is unclear because all of these bacterial species are associated with vaginal dysbiosis. The composition and significance of the intermediate Nugent category remains unclear. One molecular study suggested that this category is a transition state from a lactobacilli-dominated VMB to dysbiosis or vice versa [40], but another study found an association with VMB clusters dominated by the facultative anaerobic bacteria that have been implicated in aerobic vaginitis [4], [22]. While it is important to investigate this further to allow for the proper interpretation of epidemiological studies that have used/are using Nugent scoring to characterize the VMB, it is likely that future studies will replace Nugent scoring by molecular VMB characterization and quantification.

We found much less consensus on VMB associations with sociodemographic, behavioral, and clinical characteristics, mostly because few studies were designed to evaluate these. Three areas of consensus stood out: Vaginal colonization with *Candida* spp. was consistently more common in women with a lactobacilli-dominated VMB than women with bacterial dysbiosis [26], [31], [72], infection with *Trichomonas vaginalis* is associated with vaginal dysbiosis [64], and a high level of estradiol is consistently associated with lactobacilli [18], [59], [69], [70]. The latter is also supported by many studies that evaluated the VMB by microscopy (reviewed in [84]). The data on the associations between the VMB and HIV and HPV infection are not entirely consistent but also point in the direction of decreased lactobacilli and increased bacterial diversity when a STI is present. We recently confirmed this in a study in women at high risk of HIV and other STIs in Rwanda [85]. This study showed that women with *L. crispatus*-dominated VMB had the lowest prevalence of HIV, HPV and herpes simplex type 2 (and had no bacterial STIs), with a slight increase in women with a *L. iners*-dominated VMB, and a significant increase in women with vaginal dysbiosis. A similar trend was found for HIV-1 RNA shedding in the genital tract of HIV-positive women. Since the study was cross-sectional, the temporality of these relationships remains to be elucidated.

It is worth emphasizing that the molecular studies did not identify large VMB differences between adolescent, reproductive age, and post-menopausal women [24], [79], except in post-menopausal women with vaginal atrophy and dryness [49]. Post-menopausal women have lower estrogen levels, which might lead to less protection from dysbiosis. However, they no longer menstruate, and are therefore protected from the potentially negative effects of menstrual blood and increased vaginal pH on the VMB.

Our review also highlighted many research gaps. Most importantly, we still do not sufficiently understand how the VMB is established and maintained, and how bacterial dysbiosis develops and resolves. In particular, the roles of *L. crispatus* (which seems to inhibit dysbiosis), *L. iners* (which does not seem to inhibit dysbiosis), and *G. vaginalis* and *Prevotella* spp. (which are often present in healthy women in low abundance but greatly increase in abundance in the dysbiotic state) are not well understood. The role of *L. iners* is particularly controversial [9], [86], [87]. *L. iners* is well adapted to the vaginal niche, is present in many different types of VMBs, and often persists after antibiotic treatment. This could mean that *L. iners* easily tolerates the presence of other bacteria (which in turn could lead to dysbiosis), or that it helps to restore a lactobacilli-dominated VMB during and after dysbiosis and/or antibiotic treatment. One appealing hypothesis regarding the development of dysbiosis is the formation of a vaginal biofilm [88]. Current evidence suggests that *G. vaginalis* can be present in the vagina as dispersed bacteria or as biofilm-associated (cohesive) bacteria, with the former associated with a lower total bacterial load than the latter [89]. *In-vitro* studies suggest that when the concentration of *G. vaginalis* increases, it starts to adhere to the vaginal epithelium, providing a scaffolding to which other species adhere [90]. Initial human biopsy studies focused on *A. vaginae* as another potentially important biofilm member [91], and one study found that *L. iners* increases *G. vaginalis* adherence *in-vitro* (although this did not reach statistical significance) [92]. However, more research is needed to properly evaluate the potential role of all relevant lactobacilli and dysbiosis-associated bacteria in biofilm formation. Furthermore, it is not yet entirely clear whether the dispersed and cohesive forms of *G. vaginalis* represent different *G. vaginalis* strains. Recent studies revealed that different *G. vaginalis* strains have different metabolic and virulence properties [93], encode different types of biofilm-associated proteins [94], and behave differently in *in-vitro* biofilm experiments [95]. The biofilm hypothesis might explain the high persistence and recurrence rates of dysbiosis because bacteria in the biofilm are dormant and therefore less susceptible to antibiotics [88]. In addition, the extracellular matrix surrounding the bacteria in the biofilm inhibits penetration of lactic acid, natural antimicrobial compounds, and antibiotics [88]. However, other explanations, such as reinfection by sexual partners and spore formation, might also play a role [21]. The biofilm hypothesis might also explain why *Candida* spp. more commonly overgrow when only dispersed bacteria are present and epithelial cells are exposed: *Candida* needs to attach to epithelial cells to thrive in the vagina [96].

Whether bacterial dysbiosis is symptomatic or not most likely depends on the degree and nature of the dysbiosis, bacterial loads, type and quantity of virulence factors expressed by bacteria, and the intensity and nature of the host’s immune responses [97]. ‘Thresholds’ (in terms of bacterial loads and diversity) might exist. While most of the above-mentioned dysbiosis-associated bacteria are never pathogenic in immune-competent hosts, streptococci, staphylococci and *E. coli* can cause invasive disease when present in sufficiently high abundance. *S. agalactiae* has been particularly well studied in that regard, and studies have indeed shown that the greater the density of colonization, the greater the probability of invasive disease in postpartum women and their neonates [98]. Only three molecular studies included in our review reported VMB clusters dominated by streptococci, staphylococci, and/or *E. coli* [35], [58], [65] but many additional studies showed presence of these bacteria in low abundance; this is in agreement with studies using selective culture media [7]. *In-vitro* studies confirm that *S. agalactiae* only inhibits growth of other bacteria at concentrations higher than 10^9^ colony forming units per ml (but does not inhibit *S. aureus* and *E. coli*), and such high concentrations are rarely seen *in vivo* [98]. If aerobic vaginitis is defined as a VMB composition dominated by these bacteria, we conclude that it does exist but is not common. Future studies of invasive infections by streptococci, staphylococci, and *E. coli* should determine vaginal concentrations and not just vaginal presence.

We also do not yet sufficiently understand the metabolic synergies and dependencies of the various bacterial communities that are commonly found in the vagina. Recent studies have focused on *L. iners*, which is present in almost all women worldwide, in healthy and dysbiotic states. These studies suggest that *L. iners* is highly adapted to the vaginal compartment [9], [86], but it differentially expresses over 10% of its genome in dysbiotic compared to healthy states, with increased expression of a cytolysin, mucin, glycerol transport and related metabolic enzymes [87]. These changes likely result in the production of succinate and other short-chain fatty acids as the end product of metabolism as opposed to lactic acid, leading to an increased vaginal pH. *L. iners* might also be the first *Lactobacillus* species to recover after dysbiosis [59], which suggests a bidirectional relationship between *L. iners* and vaginal pathogens or dysbiosis. Other studies have noted synergistic effects between *G. vaginalis* and *Prevotella* spp., perhaps due to metabolic dependencies [91], [98]. At the moment, metagenomic studies of vaginal bacteria are ongoing but difficult to conduct because the public sequence databases do not yet contain all relevant bacterial genomes.

While molecular techniques have significantly improved our understanding of the VMB, some limitations should be noted. Molecular techniques detect viable as well as non-viable organisms, some cannot reliably differentiate species within a genus, some cannot adequately detect minority species, and most are not fully quantitative. We have taken this into account in our data interpretations as much as possible. Furthermore, even when the same molecular techniques were used, different laboratories used different operating procedures. Not all of these are important, but some (such as DNA extraction methods, amplification platform, choice of amplification target or of variable 16S region, choice or design of primers, and the presence or absence of proper negative controls to detect contamination) might result in significant inter-laboratory variation [14]. We were fully aware of these limitations and therefore focused this review on areas of consensus.

Now that the VMB of women with and without dysbiosis in different parts of the world have been well described, and molecular techniques have become more accessible and affordable, we believe that the time has come to incorporate these techniques into larger epidemiological studies with clinical outcomes. These studies should investigate the etiology and pathogenesis research gaps that were outlined above, but also possible transmission patterns of VMB bacteria, and the temporal relationships between the VMB and adverse reproductive health outcomes, such as HIV/STIs, pelvic inflammatory disease, adverse pregnancy outcomes, and invasive infections in pregnant/postpartum women and their neonates. At the moment, most treatment guidelines only advise clinicians to treat symptomatic vaginal dysbiosis, but this might have to be re-evaluated in specific at risk population groups (such as pregnant women or women highly exposed to HIV) if dysbiosis is identified as a strong risk factor for adverse outcomes in sufficiently powered longitudinal studies. In parallel, interventions that prevent dysbiosis, disrupt biofilms, and restore and maintain lactobacilli-dominated microbiota, should continue to be optimized and tested. The VMB studies discussed in this review have provided us with the tools to properly evaluate the safety and efficacy of such interventions. If safe, efficacious and affordable interventions are identified, they could potentially have a significant public health impact.

## Supporting Information

Checklist S1 PRISMA checklist.
(DOCX)

Diagram S1 PRISMA Flow-Diagram.
(DOC)
