# Supplementary material for: The Vaginal Microbiota: What Have We Learned after a Decade of Molecular Characterization?
Source: PLoS One. 2014 Aug 22;9(8):e105998. doi: 10.1371/journal.pone.0105998 (PMC4141851; doi:10.1371/journal.pone.0105998)
Supplement: Diagram S1 — PRISMA Flow-Diagram. (DOC) [file pone.0105998.s002.doc]

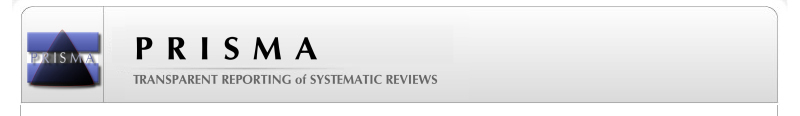
**PRISMA 2009 Flow Diagram**

**Screening**

**Included**

**Eligibility**

**Identification**

Records identified through database searching
(n =475)

Additional records identified through other sources
(n =20)

Records after duplicates removed
(n =495)

Records screened
(n =495)

Records excluded
(n = 425)

Full-text articles assessed for eligibility
(n = 70)

Full-text articles excluded, with reasons
(n = 7)

Studies included in qualitative synthesis
(n = 63)

Studies included in quantitative synthesis (meta-analysis)
N/A
